# Effect of Endotoxemia in Suckling Rats on Pancreatic Integrity and Exocrine Function in Adults: A Review Report

**DOI:** 10.1155/2018/6915059

**Published:** 2018-01-14

**Authors:** Jolanta Jaworek, Barbara Tudek, Paweł Kowalczyk, Michalina Kot, Joanna Szklarczyk, Anna Leja-Szpak, Piotr Pierzchalski, Joanna Bonior, Artur Dembiński, Piotr Ceranowicz, Zygmunt Warzecha, Katarzyna Nawrot-Porąbka, Krzysztof Gil

**Affiliations:** ^1^Department of Medical Physiology, Faculty of Health Sciences, Jagiellonian University Medical College, 12 Michałowskiego Street, 31-126 Cracow, Poland; ^2^Institute of Biochemistry and Biophysics, Polish Academy of Sciences, 5A Pawińskiego Street, 02-106 Warsaw, Poland; ^3^Department of Animal Nutrition, The Kielanowski Institute of Animal Physiology and Nutrition, Polish Academy of Sciences, 3 Instytucka Street, 05-110 Jabłonna, Poland; ^4^Department of Physiology, Faculty of Medicine, Jagiellonian University Medical College, 16 Grzegorzecka Street, 31-531 Cracow, Poland; ^5^Department of Pathophysiology, Faculty of Medical Sciences, Jagiellonian University Medical College, 18 Czysta Street, 31-121 Cracow, Poland

## Abstract

*Background*. Endotoxin (LPS), the component of Gram-negative bacteria, is responsible for sepsis and neonatal mortality, but low concentrations of LPS produced tissue protection in experimental studies. The effects of LPS applied to the suckling rats on the pancreas of adult animals have not been previously explored. We present the impact of neonatal endotoxemia on the pancreatic exocrine function and on the acute pancreatitis which has been investigated in the adult animals. Endotoxemia was induced in suckling rats by intraperitoneal application of LPS from *Escherichia coli* or *Salmonella typhi*. In the adult rats, pretreated in the early period of life with LPS, histological manifestations of acute pancreatitis have been reduced. Pancreatic weight and plasma lipase activity were decreased, and SOD concentration was reversed and accompanied by a significant reduction of lipid peroxidation products (MDA + 4 HNE) in the pancreatic tissue. In the pancreatic acini, the significant increases in protein signals for toll-like receptor 4 and for heat shock protein 60 were found. Signal for the CCK1 receptor was reduced and pancreatic secretory responses to caerulein were diminished, whereas basal enzyme secretion was unaffected. These pioneer studies have shown that exposition of suckling rats to endotoxin has an impact on the pancreas in the adult organism.

## 1. Introduction

Lipopolysaccharide (LPS, endotoxin), a major component of the outer membrane of Gram-negative bacteria, is responsible for these bacterial pathogenicities [1, 2]. LPS is a lipid-polysaccharide molecule composed of three parts: (1) lipid A, which represents a toxic element; (2) the oligosaccharide core; and (3) polysaccharide—an antigen O, highly immunogenic. This antigen constitutes 20–40 units; each unit represents 3 sugars [3, 4].

Inactive endotoxins are incorporated into the bacterial outer membrane and covered with a polysaccharide capsule [2]. Lipid A, the toxic moiety of LPS, could be released into the extracellular fluid as the result of bacterial cell damage. Free lipid A is exposed to the immune cells such as macrophages, monocytes, and polymorphonuclear leukocytes [4, 5]. Macrophages, which are activated by LPS, release cytokines and acid hydrolases and produce radical oxygen species (ROS) and nitric oxide (NO). The above substances turn on multiple mechanisms involved in the cellular defense and killing of bacteria [5–8]. Massive infection of an organism with Gram-negative bacteria is related to the release of high amount of LPS in the body fluids and might lead to the septic shock and multiple organ failure [9–12].

Endotoxin molecules are transported in the blood by specific carrier protein—LPS-binding protein (LBP). This acute-phase protein is produced in the liver and in the other tissues (kidney, heart, and lungs) in response to proinflammatory interleukins [13]. Bacterial infection stimulates the production of LPB and increases its blood level [14]. LBP conveys LPS molecule to the cell membrane, where the complex LPS-LBP binds to the CD14 receptor. This membrane receptor is devoid of intracellular activity, because of the lack of the transmembrane domain [15]. Activation of the CD14 receptor is the first step in the process of the LPS-induced signal transduction pathway. The next step involves toll-like receptor 4 (TLR4), leading finally to the mobilization of NF-*κ*B [16–18]. TLR4 receptors have been detected in the pancreas, in the acinar cells, in the pancreatic islets, and also in the vascular system [19]. Previous studies have shown that TLR4 protein was responsible for the apoptosis of pancreatic cells during acute pancreatitis [20].

The effect of LPS on the pancreas depends on their dose and on the duration of exposition. Early studies of Vaccaro et al. [21] have revealed that chronic exposition of the rats to *Salmonella typhi* endotoxin produced impairment of the pancreatic exocrine function. Also, our previous experiments have shown that prolonged administration of LPS from *E. coli* (10 mg/kg × 5 days) produced mild pancreatic necrosis [22]. To the contrary, our subsequent studies presented the beneficial effect of LPS, given at a low dose, on pancreatic inflammation. Pretreatment of the rats with a single low dose of LPS (1 mg/kg) prior to the induction of acute pancreatitis resulted in the increase in a pancreatic defense mechanism and the significant reduction of pancreatic inflammatory damage [23–25].

Chronic exposition to endotoxin could be very dangerous for the organisms, particularly in the early period of life. The development of the immune system is incomplete in the young organisms, and the responsiveness of neonates to the bacterial antigens is imperfect. Bacterial infections in the neonatal period of life could often produce sepsis with fatal outcome [12]. Massive neonatal endotoxemia could often lead to the decreased protein synthesis, tissue damage, impairment of brain functions, and sepsis with high mortality rate [26–29]. However, it was not clear how endotoxemia, which takes place in the early period of life, affects the function of the immune system and what is the impact of such intoxication on the gastrointestinal inflammation in the adult organism. We were not able to find the effect of endotoxemia induced in the suckling period of life on the pancreas of adult individuals. Because of the lack of such data, we decided to investigate the consequence of neonatal endotoxemia on the pancreatic secretion of enzymes and on acute pancreatitis in the same adult animals.

## 2. Material, Method, and Experimental Schedule of Studies on the Suckling Rats Subjected to LPS

In the next series of our studies, suckling rats (2 weeks old, weighing 30–40 g) have been employed and injected with LPS for five consecutive days. LPS, purchased from Sigma Co., which originated from *Escherichia coli* (*E. coli*) and from *Salmonella typhi* (*S. typhi*) were given to the separate groups of animals. Control rats received injections of physiological saline. LPS has been administered at various doses: 5, 10, or 15 mg/kg/day. Each group of rats received separate doses of LPS. Total doses of LPS received by each group of rats were 25, 50, or 75 mg/kg. The doses of LPS used in our study were not particularly toxic, and all young rats have survived. Three months later, the same rats, as adults, have been used for the studies on pancreatic exocrine secretory functions, in the experiments on acute pancreatitis [30, 31].

## 3. Studies on the Effect of Neonatal Endotoxemia on the Pancreatic Secretory Function in Adults

To our best knowledge, the influence of endotoxemia induced in the early period of life on the pancreatic function of the mature organism has not been previously explored. The previous study on the effect of endotoxin on the pancreatic exocrine function has been performed by Vaccaro et al. [21] on the adult rats, which have been treated with LPS at a dose of 4 mg/kg for 7 days. Such chronic application of LPS to the adult rats caused the significant reduction of their pancreatic secretory function. It has been reported in the same study that incubation of the acinar cell line AR42J with LPS resulted in the apoptosis of these cells and in the increased mRNA signals for pancreatitis-associated protein (PAP) and for proinflammatory cytokines [21].

**Figure 1:**
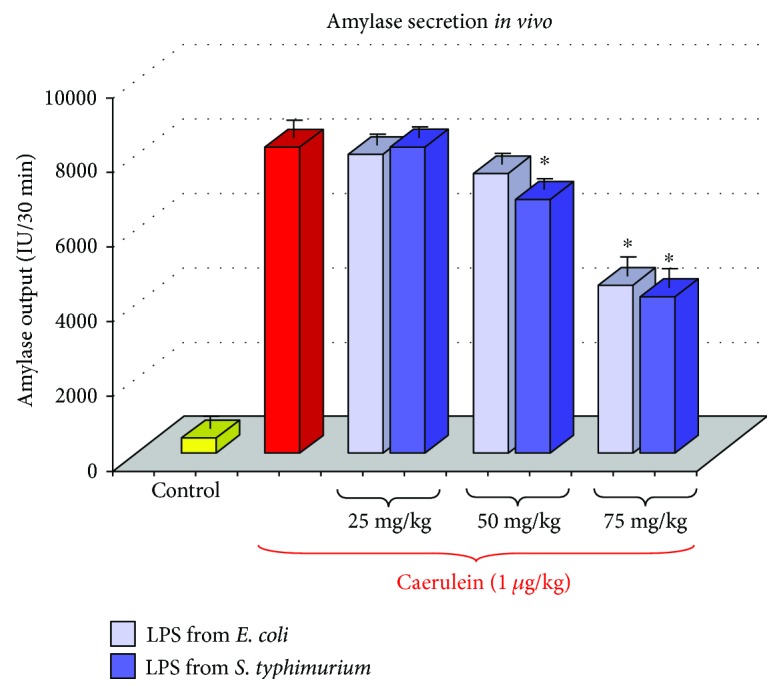
Results of an *in vivo* study—amylase responses to caerulein (1 *μ*g/kg i.p.) in adult rats, which have been subjected in the suckling period of life to endotoxin from *E. coli* or *S. typhi* given at total doses of 25, 50, or 75 mg/kg. C—normal control. Results are means ± SEM from 4 separate experiments, each performed on 6 rats. The asterisk indicates a significant (*p* < 0.05) decrease below the value obtained from rats untreated with LPS and stimulated with caerulein.

Our study failed to show any signs of inflammation in the pancreatic tissue taken from adult rats, which have been subjected to endotoxin treatment in the early period of life. In these animals, amylase blood level was not significantly different from this enzyme blood concentration measured in the control rats, untreated with LPS. Also, basal (unstimulated) secretion of amylase was not affected by neonatal endotoxemia (Figure 1).

In contrast to the unaffected basal pancreatic secretory function, we have observed that amylase secretion induced by caerulein or by diversion of pancreatic juice to the exterior (DPJ) was markedly reduced in the adult rats pretreated in the suckling period of life with LPS, as compared to the untreated control. A dose of 1 *μ*g/kg of caerulein, given to the control rats, produced amylase output reaching about 7800 IU/30 min, whereas in the adult animals pretreated with 75 mg/kg of LPS in the infancy, amylase response to1 *μ*g/kg of caerulein achieved about 4000 IU/30 min (Figure 1). The above-described reduction of pancreatic enzyme secretion was dependent on the dose of endotoxin, which has been given to the rat pups. Treatment of the rat pups with the higher dose of LPS (75 mg/kg) resulted in the strong reduction of pancreatic enzyme secretion measured in the same animals 3 months later and achieved about 50% of the control value. In the group of rats pretreated in infancy with a lower dose of LPS, which was 50 mg/kg, the amylase response to 1 *μ*g/kg of caerulein was less reduced and achieved about 70% of the control value (Figure 1) [30].

It is worth to remember that pretreatment of the rats with LPS from *E. coli* resulted in the similar reduction of the pancreatic secretory function as was observed in the rats subjected to the administration of LPS from *S. typhi* and no significant differences have been found between the both groups of rats; these rats pretreated with LPS from *E. coli* presented the same impairment of pancreatic exocrine secretion as those, which have been subjected to LPS from *S. typhi* (Figure 1).

**Figure 2:**
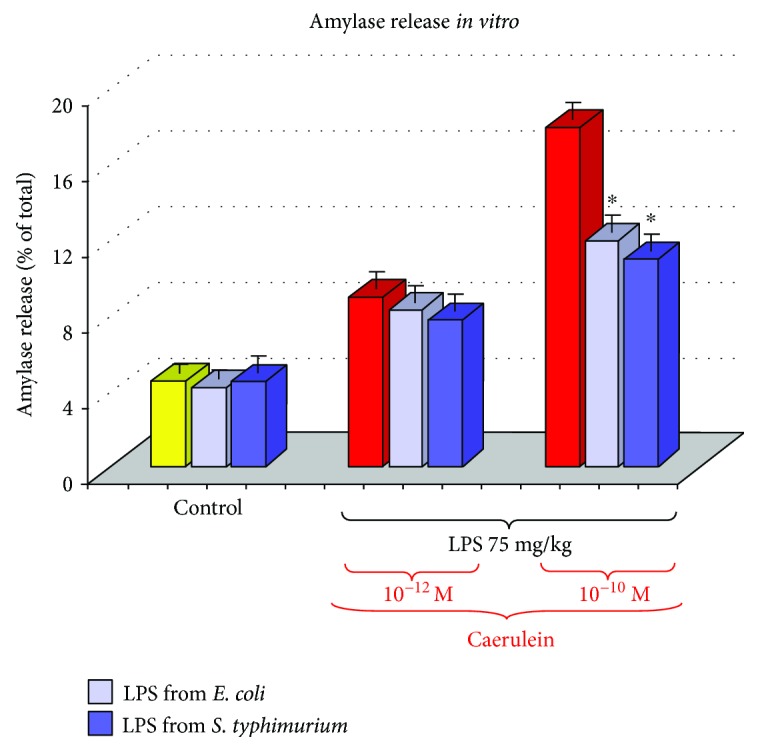
Study *in vitro*—amylase release from isolated pancreatic acini with or without caerulein stimulation. Acini were isolated from the adult rats, which have been subjected in the suckling period of life to endotoxin from *E. coli* or *S. typhi* given at a total dose of 75 mg/kg. C—control. Results are means ± SEM from 4 separate experiments, each performed in duplicate. The asterisk indicates a significant (*p* < 0.05) decrease below the value obtained from control rats untreated with LPS.

**Figure 3:**
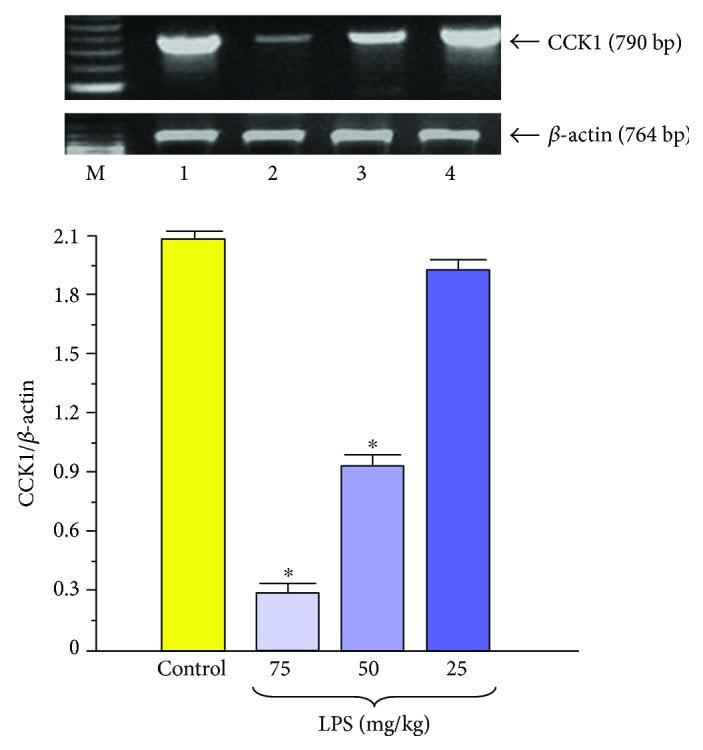
Western blot analysis of CCK1 receptor protein level in the isolated pancreatic acini obtained from the adult rats which have been subjected in the suckling period of life to endotoxin from *E. coli* given at total doses of 25, 50, or 75 mg/kg. Control—intact rats. Results are means ± SEM from 6 separate experiments. The cross indicates a significant (*p* < 0.05) decrease below the control value. The asterisk indicates a significant (*p* < 0.05) decrease below the control. The blots were stripped and probed with GAPDH to document equal protein loading. The results were obtained in 4 consecutive experiments and are representative for the observed phenomenon.

To explain the mechanism of the above reduced responsiveness of the pancreatic gland to caerulein, *in vitro* experiments on isolated pancreatic acini have been employed. In pancreatic acini, obtained from rats pretreated in the suckling period of life with LPS, pancreatic amylase release was significantly reduced as compared to the secretion of this enzyme from the control acini originating from young rats untreated with endotoxin (Figure 2). It is possible that the above-presented decreased secretory response of pancreatic acini to caerulein could be related to the changes in the CCK receptor, because the signal for the CCK1 receptor was significantly and dose-dependently reduced in the pancreatic acini obtained from adult rats injected with endotoxin in the suckling period of life (Figure 3) [31].

As the result of these pioneer experimental studies, we can conclude finally that pretreatment of the rat pups with endotoxin does not affect basal amylase secretion of adult animals *in vivo* but significantly reduced that stimulated by caerulein or diversion of pancreatic juice to the exterior. This impairment of the pancreatic exocrine secretory function could be related, at least in part, to the changes in the CCK1 receptor on pancreatic acini.

## 4. Neonatal Endotoxemia and Acute Experimental Pancreatitis in Adults

Acute pancreatitis (AP) is a serious disease, in which pathogenesis is still not clarified [32] and studies to elucidate this issue last for many years [33]. The severe form of this disease, which is complicated by sepsis, infected pancreatic necrosis, and multiple organ failure, often results in the fatal outcome [34–37]. In acute pancreatitis, systemic endotoxemia resulted from the translocation of Gram-negative bacteria and their toxins to the circulation. This translocation is facilitated by increased intestinal permeability and apoptosis of the endothelial cells [38–40]. Endotoxemia exacerbates the course of acute pancreatitis and leads to the increased rate of mortality in the severe form of this ailment [41].

In our former study, we have demonstrated that application of a low single dose of LPS prior to the induction of caerulein-induced pancreatitis made this pancreatitis less severe and markedly alleviated pancreatic inflammatory damage [23–25, 42]. Taking into consideration the harmful effect of endotoxemia on the pancreas and the protective effect of low-dose endotoxin pretreatment on acute pancreatitis, the question arises if it is possible that endotoxemia, which takes place at the early period of life, could affect the severity of acute pancreatitis induced at the adult age? Our subsequent studies on the rats have been related to this subject because such experiments have not been done before.

**Figure 4:**
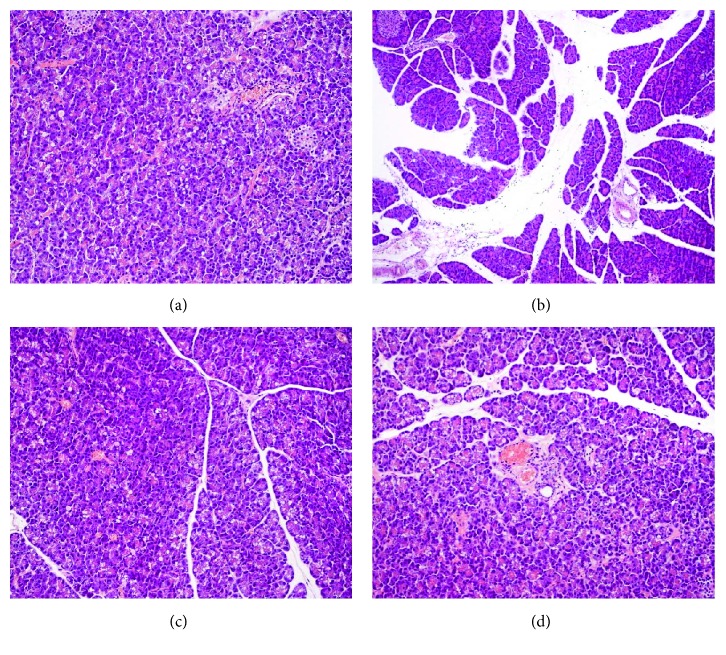
Histological picture of pancreatic tissue taken from control rats (a), from rats subjected to acute caerulein-induced pancreatitis (b), and from the animals with or without acute pancreatitis subjected in the suckling period of life to endotoxin from *E. coli* (c) or *S. typhi* (d) given at a total dose of 75 mg/kg. Hematoxylin and eosin stain.

In the adult rats, which have been pretreated with LPS in the suckling period of life at a dose of 15 mg/kg/day for 5 days, the pancreatic inflammatory changes were significantly attenuated as was shown by histological assessment (Figure 4). In these rats pretreated in the infancy with endotoxin from *E. coli* or *S. typhi*, the indicators of the severity of AP, such as amylase or lipase blood levels, were less pronounced [43].

**Figure 5:**
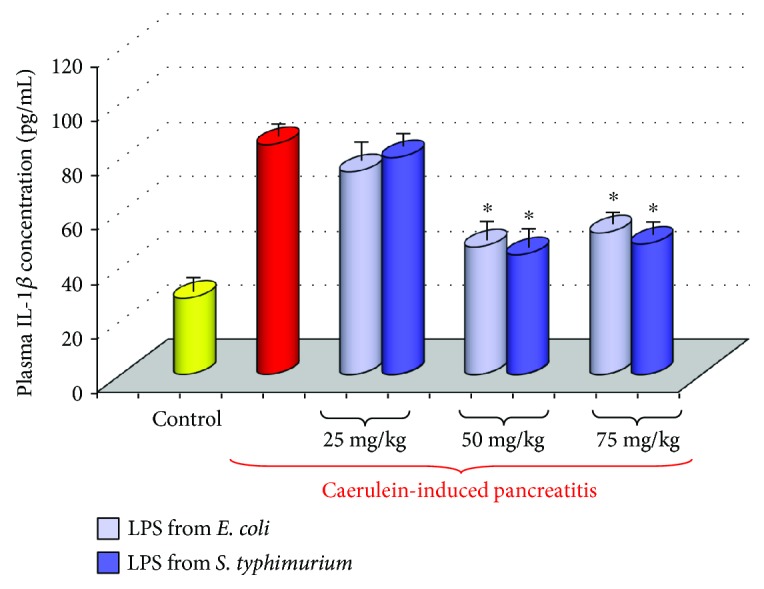
Interleukin 1*β* plasma concentration in adult rats with acute caerulein-induced pancreatitis alone and in animals, which have been subjected in the suckling period of life to endotoxin from *E. coli* or *S. typhi* given at total doses of 25, 50, or 75 mg/kg. Control—intact animals. Results are means ± SEM from 6–8 separate experiments, each performed on 6 rats. The asterisk indicates a significant (*p* < 0.05) decrease below the value obtained from rats with acute pancreatitis untreated with LPS.

**Figure 6:**
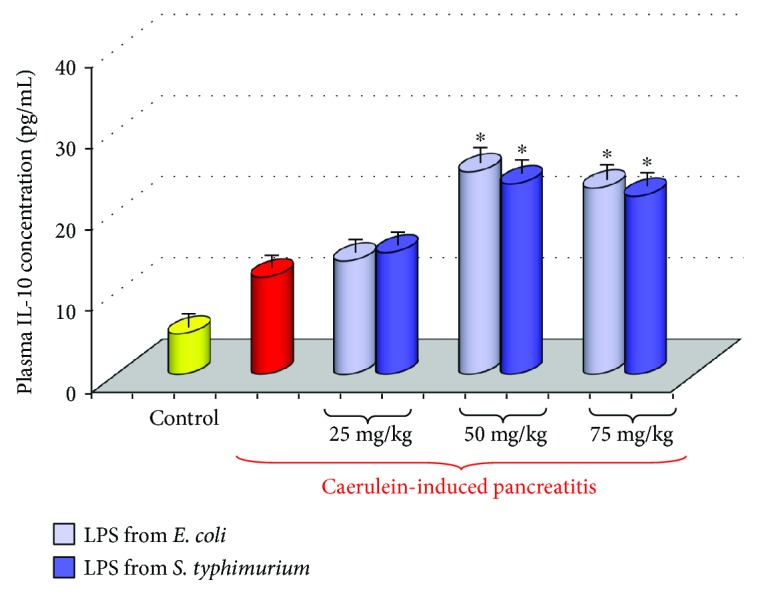
Interleukin 10 plasma concentration in adult rats with acute caerulein-induced pancreatitis alone and in animals, which have been subjected in the suckling period of life to endotoxin from *E. coli* or *S. typhi* given at total doses of 25, 50, or 75 mg/kg. Control—intact animals. Results are means ± SEM from 6 separate experiments, each performed on 6 rats. The asterisk indicates a significant (*p* < 0.05) increase above the value obtained from rats with acute pancreatitis untreated with LPS.

Activation of the immune system in the course of acute pancreatitis increases the production of proinflammatory cytokines, as well as of radical oxygen and nitrogen species (ROS and RNS), and causes the intense generation of nitric oxide (NO). These substances are among the critical factors, which are responsible for the severity of inflammation, pancreatic necrosis, and systemic complications in this disease [44–48]. High level of proinflammatory cytokines such as tumor necrosis factor *α* (TNF-*α*), interleukin 1*β* (IL-1*β*), interleukin 6 (IL-6), interleukin 8 (IL-8), or interleukin 33 (IL-33) is often related to the aggravation of acute pancreatitis [49–52]. Anti-inflammatory interleukin 10 (IL-10) alleviated the severity of this disease [53, 54]. In our study, the rise of proinflammatory IL-1*β* was significantly less abundant in the rats pretreated in the suckling period of life with LPS and subjected to acute pancreatitis at adult age, when compared to the control rats with acute pancreatitis untreated with LPS. This was accompanied by a marked increase in anti-inflammatory IL-10 in the AP rats subjected to endotoxemia in the suckling period of life. Such protection of the pancreatic gland was found in the rats subjected to LPS at higher doses (Figures 5 and 6) [43].

ROS and RNS are toxic compounds responsible for pancreatic cell damage in acute pancreatitis [47]. These substances are implicated in the development of pancreatic necrosis, septic shock, and pancreatitis-associated multiple organ dysfunction syndrome (MODS) [47, 48]. In acute pancreatitis, high amounts of ROS are produced in the immune cells infiltrating the pancreatic tissue and also in the pancreatic acinar cells [46]. ROS are responsible for the destruction of cell compartment, peroxidation of lipid membranes, and production of malondialdehyde and 4-hydroxynonenal (MDA + 4 HNE), which are commonly used as indicators of ROS production in acute pancreatitis [55–58]. Pancreatic inflammation is associated with the significant reduction of antioxidant enzymes such as superoxide dismutase (SOD), catalase (CAT), and glutathione peroxidase (GPx) [56–58]. Under normal conditions mentioned above, enzymatic antioxidants together with nonenzymatic scavengers (e.g., melatonin and vitamins E or C) protect the tissue against the noxious effects of ROS [47, 59]. Dysfunction of the scavenging system and increased production of ROS aggravated tissue inflammation and promoted leukocyte infiltration and proinflammatory cytokine generation and caused impairment of pancreatic microcirculation [47, 48].

**Figure 7:**
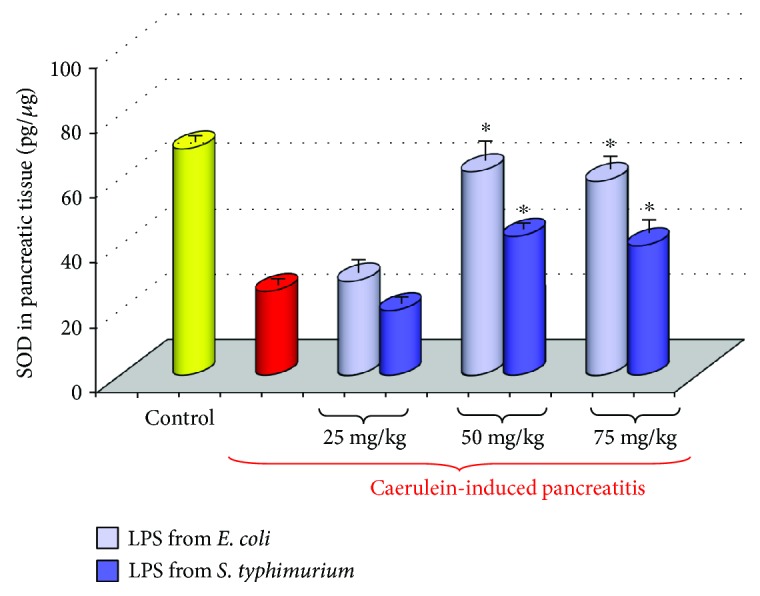
Concentration of SOD in the pancreatic tissue taken from adult rats with acute caerulein-induced pancreatitis alone and from animals, which have been subjected in the suckling period of life to endotoxin from *E. coli* or *S. typhi* given at total doses of 25, 50, or 75 mg/kg. Control—intact rats. Results are means ± SEM from 6 separate experiments, each performed on 6–8 rats. The asterisk indicates a significant (*p* < 0.05) increase above the value obtained from rats with acute pancreatitis untreated with LPS.

As we observed in our experiments, concentration of SOD, an antioxidant enzyme, in inflamed pancreatic tissue was significantly higher in the animals subjected to neonatal endotoxemia than in the rats with acute pancreatitis without such pretreatment (Figure 7). This was consistent with the observation that the rise of lipid peroxidation products was significantly lower in the pancreatic tissue taken from the rats subjected in the suckling period to endotoxemia than from the rats with acute pancreatitis without LPS pretreatment [43]. This indicates that in the animals pretreated with endotoxins in the early period of life, the antioxidant defense of pancreatic tissue was strengthened and the formation of ROS was reduced.

**Figure 8:**
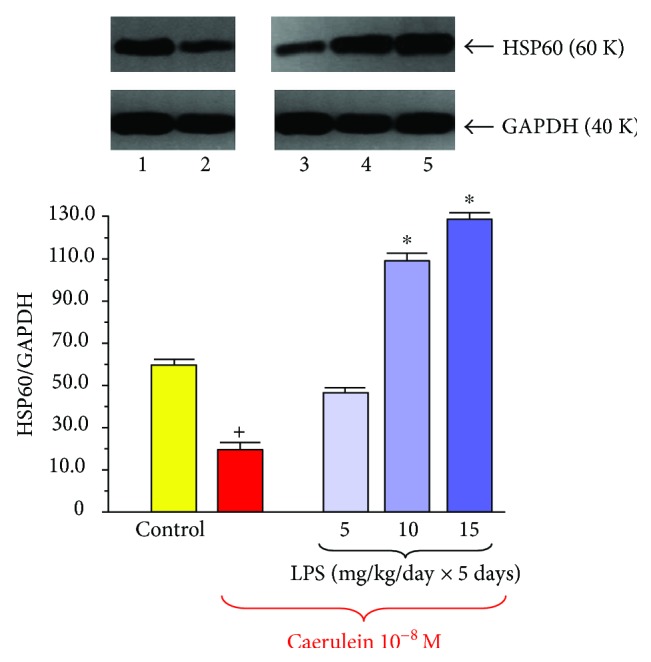
Western blot analysis of HSP60 protein level in the isolated pancreatic acini obtained from the adult rats which have been subjected in the suckling period of life to endotoxin from *E. coli* given at total doses of 25, 50, or 75 mg/kg. Control—intact rats. Results are means ± SEM from 6 separate experiments. The asterisk indicates a significant (*p* < 0.05) increase above the control value. The cross indicates a significant (*p* < 0.05) decrease below the control. The blots were stripped and probed with GAPDH to document equal protein loading. The results were obtained in 4 consecutive experiments and are representative for the observed phenomenon.

The defense mechanisms activated by neonatal endotoxemia include also the increased production of heat shock protein (HSP) in the pancreatic acini (Figure 8). HSPs are a family of polypeptides present in all cells of organisms. They are responsible for correct folding of proteins, for their transport into subcellular compartment, and for modulation of immune activity. HSPs are best known as chaperon substances effectively protecting the cell compartment against the stress-induced damage [60–62]. Exposition of cells to high temperature, to oxidative stress, or to other stressful conditions leads to the upregulation of HSP signals and to the increase in these proteins' synthesis [62, 63]. HSPs create an effective mechanism of cell defense against inflammatory damage, and induction of HSP60 or HSP70 protected against acinar cell injury in acute pancreatitis [61, 62]. The beneficial effect of HSP on acute pancreatitis has been supported by the studies on transgenic mice, showing that in animals with increased expression of HSP70, the severity of acute pancreatitis was reduced, whereas in mice devoid of HSP, this severity was markedly enhanced [64, 65]. It was also shown that HSP accelerated the recovery from acute pancreatitis [66].

Our studies on the pancreatic cell lines, AR42J and PANC-1, presented the evidence that cell protection afforded by melatonin, kynuramines, or leptin is related to the increased signal of HSP in pancreatic acinar cells [67–69]. Controversial effects of HSP have been reported in pancreatic tumor. Increased expression of HSP27 in tumor cells has been associated with increased resistance to chemotherapy and poorer prognosis in patients with pancreatic cancer [70]. In contrast to the above report, recent publication has demonstrated that higher expression of HSP27 was correlated with better patient survival [71].

HSP60 is recognized as chaperon protein preventing acinar cells from damage [62]. We have observed that in the pancreatic acini taken from adult rats subjected to endotoxemia in the suckling period of life, the signal for HSP60 protein was significantly stronger than that in the acini obtained from the rats, which have not been subjected to LPS treatment [72]. This observation strongly suggests that this increased ability to synthetize HSP60 could be the part of acinar cell resistance against the inflammatory damage in rats pretreated in infancy with LPS.

**Figure 9:**
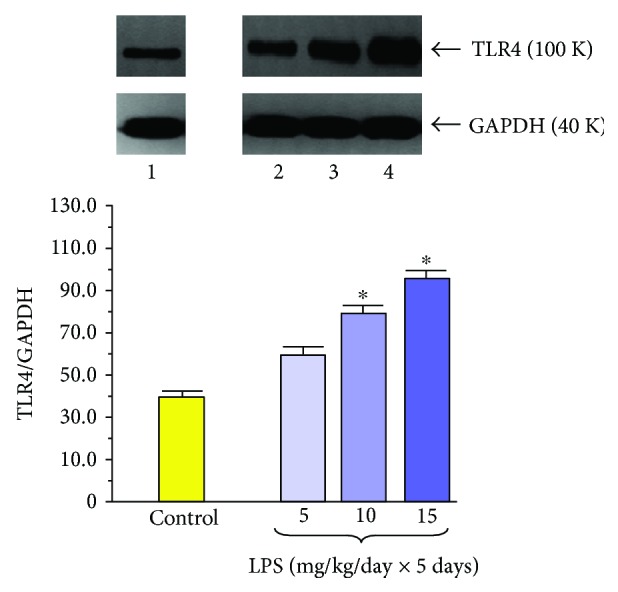
Western blot analysis of TLR4 protein level in the isolated pancreatic acini obtained from the adult rats which have been subjected in the suckling period of life to endotoxin from *E. coli* given at total doses of 25, 50, or 75 mg/kg. Control—intact rats. Results are means ± SEM from 6 separate experiments. The asterisk indicates a significant (*p* < 0.05) increase above the control value. The cross indicates a significant (*p* < 0.05) decrease below the control. The blots were stripped and probed with GAPDH to document equal protein loading. The results were obtained in 4 consecutive experiments and are representative for the observed phenomenon.

Another interesting phenomenon that was observed in the pancreatic acinar cells isolated from rats subjected to LPS in the suckling period of life presented the increased signal for toll-like receptor 4 (TLR4) (Figure 9). TLR4, which is present on the inflammatory cells, plays an important role in the innate immunity, triggering the inflammatory response through the activation of the NF-*κ*B pathway [73]. In the pancreas, TLR4 has been detected in the pancreatic ductal, acinar, and endothelial cells and involved in the induction of cell apoptosis [74]. TLR4 signal has been upregulated in acute pancreatic inflammation and perhaps is involved in the control of pancreatic cell apoptosis during the early stages of acute pancreatitis [75]. Recent *in vitro* studies have shown that neutralization of TLR4 reduced apoptosis of taurocholate-treated mouse acinar cells through inhibition of cytochrome c release and promoted these cells' viability. It was suggested that such blockade of TLR4 activity could exert protective effects on an *in vitro* model of acute pancreatitis [76]. Compatible results have been shown by Xue and Habtezion [77] who demonstrated that blockade of TLR4 on macrophages can ameliorate acute pancreatitis. Yet opposing observations concerning the consequence of apoptosis in acute pancreatitis have been presented in other recent publications. In the experimental studies on rat acute pancreatitis, it was shown that pancreatic protection was related to the activation of proapoptotic Bax and the reduction of antiapoptotic Bcl proteins leading to the activation of the final executor of apoptosis, the active enzyme caspase-3 [78]. Also in the studies on pancreatic acini subjected to bile acids, apoptosis was shown as a protective process leading to the improvement of pancreatic defense and to the restriction of the inflammatory process in the pancreas. Apoptotic programmed cell death prevents the cell membrane from interruption and from the release of lysosomal enzymes from the acinar cells, and in this way, inflammation is limited and tissues are protected from the injury [79–82].

We have found that the signal for TLR4 in the pancreatic acini taken from the rats subjected in the suckling period of life to LPS treatment was markedly increased [81]. Such abundance of TLR4 protein expression might suggest that during acute pancreatitis, the apoptotic process could be facilitated in these rats and that treatment with LPS in the early period of life enables the pancreatic cell to activate the apoptosis signaling pathway. It is likely that in LPS-pretreated rats, predominance of apoptosis over necrosis could reduce pancreatic tissue damage and might be responsible, at least in part, for amelioration of toxic inflammatory mediators and attenuation of acute pancreatitis severity [81, 82].

We can conclude that increased pancreatic defense in the rats, subjected to endotoxemia in the early period of life, resulted from several protective mechanisms such as (1) modulation of the immune system, reduction of proinflammatory cytokine production, and rise of anti-inflammatory cytokine production; (2) augmentation of the antioxidant enzyme SOD in the pancreatic tissue and decreased formation of ROS; (3) stimulation of the chaperon protein HSP60 in the pancreas; (4) stimulation of protein signal for TLR4 and subsequent activation of apoptosis in the pancreatic tissue; and perhaps (5) the reduced ability of the pancreatic gland to secrete the digestive enzymes and thus to decrease the crucial mechanism of pancreatic autodigestion.

## 5. Conclusion

Under physiological conditions, the immune cells are continuously exposed to the low amounts of LPS, which are derived from the gastrointestinal bacteria. This stimulation may be essential to maintain the certain level of attentiveness of the immune system without causing a disease. Neonatal endotoxemia affects the ability of the immune cells to produce the cytokines and increases the resistance of the organism to pancreatic inflammation. However, this endotoxemia could also turn on the impairment of the pancreatic exocrine function.
